# Effects of Social Information on the Release and Expression of Gonadotropin-Inhibitory Hormone in Birds

**DOI:** 10.3389/fendo.2019.00243

**Published:** 2019-04-24

**Authors:** Yasuko Tobari, Kazuyoshi Tsutsui

**Affiliations:** ^1^Laboratory of Animal Genetics and Breeding, Department of Animal Science and Biotechnology, School of Veterinary Medicine, Azabu University, Sagamihara, Japan; ^2^Laboratory of Integrative Brain Sciences, Department of Biology, Waseda University, Tokyo, Japan

**Keywords:** gonadotropin-inhibitory hormone, gonadotropin-releasing hormone-I, hypothalamus–pituitary–gonadal axis, neuropeptide, noradrenaline, RFamide-related peptide

## Abstract

The social environment changes circulating hormone levels and associated behavior in animals. Although social information is perceived by sensory systems in the brain, and peripheral reproductive hormonal levels are regulated mainly by the hypothalamus–pituitary–gonadal (HPG) axis, the neurochemical systems that convey social information to the HPG axis were not well-understood until the 2000s. In recent years, a growing body of evidence has demonstrated that a neuropeptide localized in the hypothalamus, gonadotropin-inhibitory hormone (GnIH), is responsive to social information. GnIH was first identified in the quail hypothalamo-hypophyseal system and named for its ability to inhibit gonadotropin secretion. Hypothalamic GnIH neurons have thus begun to be regarded as integrators, translating social information into changes in the levels of circulating gonadal hormones through the HPG axis. Here, we review current research investigating the responses of the GnIH neuronal systems to social status, offspring, and the presence/absence of conspecifics, and describe the neurochemical pathways linking visual perception of a potential mate to a rapid change in blood gonadotropin levels via the hypothalamus–pituitary axis in male birds.

## Introduction

Many environmental factors can influence reproductive physiology in animals. While photoperiodic changes can induce or suppress reproductive activity in some species, additional environmental signals, such as temperature, humidity, food availability, and the presence of conspecifics, are also important in fine-tuning the timing of breeding (1). Social animals receive specific signals from the presence of conspecifics, and these signals evoke changes in neuropeptide systems, appropriate behavioral, and physiological responses for breeding (2–5).

It is now understood that social interactions influence neuropeptide systems. Neurons in the bed nucleus of the stria terminalis, which produce the avian nonapeptide arginine vasotocin, are selectively responsive to social stimuli and promote preferences for larger flock sizes in gregarious finches (6–8). Numbers of activated parvocellular neurons expressing mesotocin, within the paraventricular nucleus are correlated with courtship frequency in adult male brown anoles (*Anolis sagrei*) (9). In male European starlings (*Sturnus vulgaris*), only males that acquire nesting sites display high rates of sexual and agonistic behavior. Neurotensin (NT) is a neuropeptide implicated in motivation. NT expression in the medial preoptic nucleus (POM) is positively associated with courtship behavior in male starlings (10). Meanwhile male starlings with nesting sites have lower kappa-opioid receptor (opkr1) expression in the POM, and opkr1 expression is negatively associated with courtship vocal behavior (11). These studies suggest neuropeptide responses to social interaction may be related to expression of social behaviors.

The courtship behavior of male birds is a good model for studying the neural mechanisms that provoke hormonal responses to specific cues from conspecifics. When viewing a female, male birds show rapid changes in blood androgen concentrations. This social modulation of the concentrations of blood androgens may be a mechanism for adjusting androgen-dependent behaviors to the current social environment (12).

Testicular androgen production and release are under the control of the hypothalamus–pituitary–gonadal (HPG) axis (13). The hypothalamic neuropeptide gonadotropin-inhibitory hormone (GnIH) acts on the HPG axis. A growing body of research shows that hypothalamic GnIH neurons are responsive to social information. Therefore this hypothalamic neuropeptide is viewed as an integrator molecule that translates social environmental information into reproductive physiology (14). In this review, we summarize current research on the social modulation of the release and expression of GnIH in the brain, with particular reference to GnIH responses to conspecifics.

## Hypothalamic Neuropeptide-Related Avian Reproduction

Avian reproductive physiology and blood androgen concentrations are regulated by the HPG axis. Gonadotropin-releasing hormone-I (GnRH1) is an evolutionarily conserved decapeptide and the central neuroendocrine regulator of the HPG axis (15). In stimulatory conditions, GnRH1 is released from nerve axons in the median eminence (ME) of the hypothalamus, stimulating the production and release of luteinizing hormone (LH) and follicle-stimulating hormone from the anterior pituitary gland, which, in turn, regulate testicular and ovarian functions (16, 17). In addition, GnIH has been revealed to be a pathway for reproductive control (18–20). GnIH was first identified in the quail hypothalamo–hypophyseal system and named for its ability to inhibit gonadotropin secretion (21). GnIH may also decrease GnRH1 synthesis and release in birds, because GnIH neurons make contact with both GnRH1 cell bodies and their nerve terminals in house sparrows (*Passer domesticus*) and European starlings (22, 23). GnIH receptors have been identified in the chicken and quail pituitary (24, 25), and are also expressed in GnRH1 neurons in the starling brain (23). These findings suggest that, as well as acting on the pituitary gland, GnIH acts on GnRH1 neurons to inhibit gonadotropin release.

Social information is perceived by the brain via sensory systems. The information seems to converge on the hypothalamus and is then transduced into a reproductive endocrine response through the portal vessels to the adenohypophysis (26). In male ring doves (*Streptopelia risoria*), synthesis of GnRH1 mRNA, and protein in the preoptic area of the hypothalamus increases in response to a 1–2-h courtship interaction with a female (3), prior to an increase in blood LH levels (27). In female white-throated sparrows (*Zonotrichia albicollis*), hearing courtship songs for 42 min induces an increase in the expression of immediate early genes in the mediobasal hypothalamus, which is the avian brain region involved in the control of GnRH1 release and elevation of blood LH level (28).

Recent studies have indicated that avian GnIH neurons in the hypothalamus are influenced by environmental information, including photoperiod and stress (29–31). GnIH has begun to be regarded as a modulator of reproductive activity in response to the external and internal environment (18–20, 32). In the 2010s, some research groups began to examine changes in the activity of GnIH neurons of avian species following various social interactions.

## Effects of Social Status and Breeding Condition on GnIH Expression

European starlings are cavity nesters and exhibit biparental care, with males and females sharing incubation and provisioning duties. To examine whether social status can alter hypothalamic GnIH synthesis to modulate reproductive physiology in different stages of the breeding season, Calisi et al. (33) manipulated nesting opportunities for pairs of European starlings and examined changes in the hypothalamic GnIH synthesis in nest owners (winners) and floaters (losers). Nest owners had significantly lower numbers of GnIH-producing cells than floaters at the beginning of the breeding season, whereas the number of GnIH-producing cells was greater in nest owners than in floaters in the middle of the breeding season. The number of GnRH1-producing cells and the levels of plasma testosterone and corticosterone did not vary with nest box ownership. Meanwhile only European starlings that acquire nesting sites display high rates of reproductive behaviors including sexual and agonistic behavior. Maney et al. (34) reported that GnRH2 enhances copulation solicitation in estrogen-implanted female white-crowned sparrows (*Zonotrichia leucophrys gambelii*) exposed to the song of males. Centrally administered GnIH rapidly inhibited this reproductive behavior in estradiol-treated female sparrows (35). GnIH neurons terminate in the close proximity of GnRH2 neurons in the midbrain of sparrows and starlings (22, 23) and GnRH2 neurons express GnIH receptor mRNA in European starlings (23). In sum, these data suggest that hypothalamic GnIH neurons in starlings may modulate reproductive behaviors via direct actions on the GnRH2 system, without altering the activities of the HPG axis, in response to nest competition.

## Effects of Offspring on GnIH Synthesis

Calisi et al. (36) continued their investigations of environmental influence on GnIH-producing cells and examined the effects of reproductive stage on GnIH cell abundance in the hypothalamus of European starlings. They quantified the number of GnIH-producing cells in starlings prior to nesting, prior to incubation, at the beginning and end of incubation, and at the start of chick care. GnIH-producing cells were most abundant when birds first began to incubate their eggs, and when chicks hatched. The starlings' blood testosterone increased at the beginning of the breeding season and peaked during the nest-building and fertile period when males were defending nest boxes, attracting females, and guarding their mates. Testosterone gradually decreased during the parental stage to facilitate parental behaviors (37). These data suggest that the presence of eggs increases GnIH neuronal activity at the beginning of incubation, resulting in the suppression of circulating testosterone that stimulates a behavioral switch from aggressive and sexual behavior to parental behavior.

In addition to characterizing GnIH cell abundance over the reproductive cycle in starlings, Calisi et al. (36) removed eggs on Day 8 of incubation to examine how unpredictable events in their incubation phase could affect hypothalamic GnIH system. They expected a decrease in GnIH cell abundance would be most likely to facilitate reproductive activity in order to obtain new offspring. However, GnIH cell abundance increased as a result of egg removal. GnIH expression can increase in response to acute stress in seasonally breeding birds (30). Thus, in this case, perhaps an increase in GnIH neuronal activity after nest predation (egg removal) may stimulate parent birds to switch nest-site and this switch may be important for reproductive success of starlings.

## Effects of Female Conspecifics on GnIH Systems in Males

A longstanding goal of avian endocrine research has been to clarify how the social environment might change gonadotropin and androgen levels in birds. In Japanese quail (*Coturnix japonica*), sexually active males exhibit a transient decline in circulating testosterone when they recognize a female conspecific (38, 39), suppressing courtship vocalization and actively approaching her for copulation (5, 40–42). Using this interesting animal model, we investigated the neurochemical pathways leading from the visual perception of a female conspecific to a change in blood gonadotropin level in male Japanese quail. Male birds viewing a female had increased levels of GnIH mRNA expression in the paraventricular nucleus (PVN) of the hypothalamus, which was associated with a decrease in the blood LH level (43). Likewise, noradrenaline (NA) release increased rapidly in the male PVN immediately after viewing a female. The expression level of GnRH1 mRNA did not vary. Viewing a male conspecific did not increase the level of GnIH mRNA in male birds. In addition, we showed that NA application dose-dependently stimulated GnIH release from the diencephalic explants *in vitro*, and intracerebroventricular injection of NA reduced blood LH concentrations (43). GnIH neurons express the α2A-adrenergic receptor subtype mRNA and receive noradrenergic innervation (43). Accordingly, these findings suggest that viewing a female conspecific provokes an increase in NA release in the PVN, which directly stimulates the release of GnIH, resulting in the suppression of LH secretion from the pituitary in male quail. This NA–GnIH system is a novel neurochemical pathway through which the social milieu can rapidly influence reproductive physiology in birds.

## Effects of NA and Social Isolation on GnIH Expression

We have shown that NA treatment stimulates GnIH release from diencephalic tissue blocks (43). However, at the time of that study, it was unknown whether noradrenergic transmission plays an important role in regulating GnIH gene expression in the brain. There are two groups of noradrenergic cells in the avian brain: the locus coeruleus (LoC) group and the lateral tegmental (LT) group, whose cells of origin are diffusely distributed in the caudal pons and rostral medulla (44–46). The quail hypothalamus receives dense noradrenergic innervation (44) and contains a high concentration of NA compared to other areas of the brain (47, 48). Ligand binding experiments and *in situ* hybridization revealed a high density of α2A- and α2C-adrenergic receptors in the PVN (43, 49).

*N*-(2-chloroethyl)-*N*-ethyl-2-bromobenzylaminehydrochloride (DSP-4) causes a selective decrease in noradrenergic neuronal activity, with degeneration of the axon terminals of noradrenergic neurons originating from the LoC (50, 51). In quail, DSP-4 decreases NA concentrations only in the hypothalamus (48). To evaluate the specific role of noradrenergic LoC neurons in regulating hypothalamic GnIH gene expression, we investigated the change in GnIH gene expression in the hypothalamus of male quail in response to the decrease in hypothalamic NA by DSP-4 treatment. Interestingly, decreasing hypothalamic NA levels had different effects on brain GnIH gene expression according to the social situation of male quail. Noradrenergic ablation with DSP-4 did not change GnIH gene expression in the brains of male quail housed in an aviary, where all birds had visual and auditory (but no tactile) contact with other male conspecifics (52) or were exposed to a sexually active female (unpublished data). To remove the effect of conspecifics, after lowering diencephalic NA with DSP-4, we kept male quail alone in an empty room for 1 h. Social isolation after lowering diencephalic NA concentration increased GnIH gene expression in the brains of male quail, suggesting that noradrenergic LoC neurons have an inhibitory effect on GnIH gene expression when male quail are alone (52). Meanwhile, DSP-4 treatment did not change the plasma LH level of male quail exposed to social isolation or a female for 1 h (unpublished data), suggesting that noradrenergic LoC neurons do not influence GnIH release.

Decreasing NA secretion pharmacologically influences GnIH gene expression differently in the brains of male quail housed in an aviary vs. socially isolated quail. In the aviary, quail were housed in adjacent individual cages. The quail in the aviary exhibited strongly intermittent pecking-like behavior toward male birds in neighboring cages. On the other hand, socially isolated quail exhibited locomotion (walk, run, jump) and resting (stand, sit) behaviors. Edens (53) indicated that aggressive pecking behavior is associated with increased brain NA concentrations in male quail housed in male pairs. Additionally, the GnIH concentration in the hypothalamus is associated with the frequency of sociosexual behaviors in male quail, including pecking (54). Visual and auditory information from male conspecifics, and pecking-like behavior toward them, may induce intermittent NA release from noradrenergic axon terminals unaffected by DSP-4. This intermittent NA release may have maintained extracellular NA levels in the PVN of quail in the aviary, resulting in no difference in hypothalamic GnIH gene expression between the DSP-4 treated and control birds in the aviary.

## Effects of Social Rank on GnIH Synthesis in a Eusocial Mammal

Moving away from birds, the naked mole rat (NMR; *Heterocephalus glaber*) is a powerful model system for studying social reproductive repression, because NMRs are eusocial subterranean rodents and establish colonies wherein breeding is usually monopolized by only one female (the queen) and one to three males (dominants); the remaining colony members serve the colony as social subordinates and remain in a juvenile-like prepubescent state. Male and female subordinates are capable of transitioning to breeding status following the removal of dominants or separation from the colony. Dominants have high progesterone concentrations compared to colony-housed subordinates, and exhibit sexual behaviors. To clarify the role of RF-amide-related peptide-3 (RFRP-3, a mammalian ortholog of avian GnIH) in social regulation of puberty onset in NMRs, Peragine et al. (55) examined whether the social and reproductive hierarchy can alter hypothalamic RFRP-3 systems, and whether RFRP-3 can suppress sexual maturation in NMRs. Hypothalamic RFRP-3 expression was affected by reproductive status in NMRs. Subordinates had enhanced RFRP-3 immunoreactivity compared to dominants. Intracerebroventricular injection of RFRP-3 reduced the blood progesterone concentration and sexual interest in sexually active NMRs separated from the colony. These data suggest that hypothalamic RFRP-3 neurons in NMRs may serve as gatekeepers of puberty onset according to social rank.

## Conclusion and Future Directions

GnIH is a hypothalamic neuropeptide that suppresses and finely tunes reproduction in birds by directly modifying GnRH1 and gonadotrophin release. GnIH expression and synthesis are influenced by social status, breeding condition, and the presence of conspecifics. Female presence stimulates GnIH release via NA release in male birds. These findings suggest that GnIH neurons modulate the activities of the HPG axis in response to the social environment, as well as photoperiod and stress. Thus GnIH is a newly discovered integrator of environmental stimuli for reproduction; nevertheless, there remain unanswered questions about its roles and mechanisms of action.

First, does GnIH also play a role in rapid regulating social behaviors in response to social stimuli? Male quail increase GnIH neuronal activity when they recognize an attractive female conspecific. In addition, female presence suppresses instantly male courtship vocalization and promotes male approach for copulation. If GnIH can act directly within the brain to control reproductive behaviors (35, 54), activated GnIH neurons by female presence in male quail may modulate the neurol circuitry underlying social behaviors to change the pattern of courtship vocalization and social proximity behavior. The neural mechanisms of GnIH action in the brain regulating vocalization and social proximity behavior will be the focus of future studies.

A second question is whether NA is involved in regulating seasonal changes in GnIH function. Matt et al. (56) reported that the NA turnover rate was significantly lower in the preoptic area (the site of GnRH1 cell bodies) and the ME (the site of GnRH1 and GnIH terminals) of photorefractory white-crowned sparrows compared to photosensitive and photostimulated birds. In addition, NA is involved in the regulation of biosynthesis and release of melatonin (57), which is a potent regulator of GnIH. These data suggest that NA also plays a role in regulating seasonal changes in GnIH synthesis and release. A third question is: What is the role of noradrenergic LT neurons in social modulation of GnIH neurons? Because the hypothalamus has rich noradrenergic innervation from the LT noradrenergic systems in rodents, it is likely that the avian hypothalamus is also richly innervated by noradrenergic LT neurons. Whether noradrenergic LT neurons affect GnIH neuronal activity remains unclear, and requires further investigation.

The role of GnIH in modulating the reproductive axis in vertebrates has been well-documented. However, studies on GnIH responses to social stimuli have only been conducted in a small number of species. Clarifying what kind of social information can affect GnIH neuronal activity, and the neural and molecular mechanisms by which social information affects GnIH neurons in many kinds of animals, would contribute not only to the field of reproductive endocrinology but also the neuroscience of sociality.

## Author Contributions

All authors listed have made a substantial, direct and intellectual contribution to the work, and approved it for publication.

### Conflict of Interest Statement

The authors declare that the research was conducted in the absence of any commercial or financial relationships that could be construed as a potential conflict of interest.
